# Clinical Features and Outcomes of Acute Chlorine Gas Inhalation; a Brief Report

**DOI:** 10.22037/aaem.v10i1.1448

**Published:** 2022-02-14

**Authors:** Taymmia Ejaz, Sheema Saadia, Safia Akhlaq, Adil Aziz, Muhammad Arslan Ahmed, Aisha Fareed Siddiqui

**Affiliations:** 1 Department of Medicine, The Aga Khan University Hospital, Karachi, Pakistan.

**Keywords:** Inhalation exposure, poisoning, accidents, occupational, chlorine, gas poisoning, outcome assessment, health care

## Abstract

**Introduction:**

On March 6^th^,2020, chlorine gas leak was reported at Engro Polymer & Chemicals Plant in Karachi City, Pakistan. This study aimed to evaluate the clinical features and outcomes of patients who presented to emergency department (ED) following this event.

**Methods:**

This retrospective cross-sectional study, evaluated the clinical features and outcomes (length of hospital stay, complications, and mechanical ventilation requirement) of patients presenting to ED of Aga Khan University Hospital, Karachi, Pakistan, with history of chlorine gas exposure at the Engro Plant from 6^th^ March to 14^th^ March 2020.

**Results:**

38 patients with mean age of 33.1 ± 8.1 years presented to ED with history of chlorine gas exposure (100% male). 4 (10.5%) cases had comorbid diseases. Most common presenting symptom was dyspnea, observed in 33 (86.8%) cases, followed by cough, seen in 27 (71.1%) subjects. 13.2% (5/38) patients had infiltration on chest x-ray and 33 (86.8 %) required hospitalization. 6 (15.8%) patients had repeat presentation requiring hospitalization or ED visit. 18 (47.4%) were managed with high flow oxygen therapy, 9 (23.7%) required non-invasive ventilation and one patient was intubated due to development of pneumo-mediastinum. Mean length of stay was 1.55 ± 1.58 days and no patients died. Presence of tachycardia was the only finding significantly associated with need for oxygen (p = 0.033) and non-invasive ventilation (p = 0.012).

**Conclusion:**

The majority of patients presenting with acute chlorine gas exposure showed good clinical outcomes and rapid recovery, however, a high index of suspicion and vigilance should be maintained for complications such as pneumomediastinum and acute respiratory distress syndrome in these patients.

## 1. Introduction:

Chlorine gas, a simple halogen irritant, is available worldwide as a household and industrial chemical. Through oxidation and free radical formation (1) halogen pulmonary irritants (HPIs) cause respiratory mucosal damages, and concomitant ophthalmological and dermatological manifestations(2). Manifestations are dose-dependent, with mucosal membrane irritation occurring at concentrations less than 15 ppm, milder respiratory clinical features at concentrations more than 30 ppm, and pneumonitis and pulmonary edema at concentrations greater than 50 ppm. Death results within few to 30 minutes in exposures with 400 ppm and over 1000 ppm concentrations, respectively(2). Exposure can result from multiple sources, which include industrial accidents, inappropriate use of household cleaning agents, swimming pool chlorination accidents, burning of solid chlorine products, and deliberate release as a chemical weapon such as in World War One and Syrian Conflict(3). Health effects of chlorine gas inhalation vary based on age, comorbid conditions, exposure duration, and various other factors(3, 4). Inhalational injury results in acute short-term clinical features and long-term changes in lung function, which manifest as reactive airway dysfunction(5, 6). Industrial accidents resulting in large scale chlorine exposure incidents have occurred both in the developed and developing world(7-12). On March 6^th^, 2020, chlorine gas leak was reported at Engro Polymer & Chemicals Plant in Karachi City, Pakistan(13). Over one hundred workers were reported to have been exposed and were taken to different health care facilities in Karachi; some were brought to Aga Khan University Hospital for further management. This study aimed to evaluate clinical features and outcomes of patients who were accidentally exposed to chlorine gas in the industrial accident.

## 2. Methods:

***2.1 Study design and setting***

This study is a retrospective cross-sectional study conducted in Aga Khan University Hospital, Karachi, Pakistan, from March - April 2020. Ethical approval was taken from Institutional Ethics Review Committee Aga Khan University Hospital (ERC:2020-4918-10961). 

2.2 Participants

All patients with history of chlorine gas exposure at the Engro Plant from 6^th^ March to 14^th^ March 2020 presenting to Emergency department and those hospitalized in Aga Khan University Hospital, Karachi, Pakistan were included in the study. Patients were enrolled based on consecutive non-probability sampling. Patients with incomplete records were excluded. Diagnosis of chlorine poisoning was made based on exposure history, clinical presentation, and laboratory and radiological findings. 

***2.3 Data collection***

Data collection was done using a pre-designed proforma. Data was collected on demographic profile, co-morbid conditions, presenting clinical features and other symptoms, examination findings, oxygen requirement, laboratory finding, imaging findings, and management. Retrospective review of files and electronic health records was done. Patients who had not undergone chest x-rays or arterial blood gas tests were categorized in missing data. Length of hospital stay, complications, and mechanical ventilation requirement were considered as outcomes. 

***2.4 Statistical analysis***

Data analysis was done in SPSS Version 23. Categorical data were reported with frequencies and percentages. Continuous data were reported as mean and standard deviation. Normality tests were done to assess normality and parametric or nonparametric tests were used accordingly. Fischer’s exact and chi-square tests were done to compare categorical data, and student t-test was done to compare quantitative data. A p-value < 0.05 was considered significant. 

## 3. Results:

38 patients with the mean age of 33.1 ± 8.1 (range: 21 - 55) years presented to emergency department with history of chlorine gas exposure during the defined study period (100% male). 4 (10.5%) patients had comorbid conditions (3 hypertensions, 2 diabetes mellitus, and 1 ischemic heart disease). 

Table 1 summarizes the clinical and laboratory findings of studied cases. Most common presenting symptom was dyspnea, observed in 33 (86.8%) cases, followed by cough, seen in 27 (71.1%). Among the unusual presenting features, pre-syncope/syncope was reported by three patients and loose stools were reported by one patient.

Chest x-rays were done in 32 (84.2%) patients; among these patients 5/32 patients (15.6%) had abnormalities (pulmonary edema in 2 patients, pneumomediastinum and subcutaneous emphysema in one patient, bilateral inhomogeneous consolidations in one patient and infiltrates in right infra-hilar region in one patient).

Table 2 shows the management and outcomes of the cases. 33(86%) patients required hospitalization and 6 (15.8%) patients had repeat presentations requiring hospitalization or emergency department visit. 18 (47.4%) were managed with high flow oxygen therapy, 9 (23.7%) required non-invasive ventilation, and one patient was intubated due to development of pneumomediastinum on repeat presentation to emergency department. Mean length of stay was 1.55 ± 1.58 days and no patients died. Tachycardia was the only finding significantly associated with need for oxygen supplement (p = 0.033) and non-invasive ventilation (NIV) requirement (p = 0.012; table 3).

## 4. Discussion:

This study describes the presenting features, outcomes, management, and complications observed in patients after an industrial disaster resulting in accidental exposure to chlorine. The proportion of presenting affected population was significantly lower than that observed in other larger-scale disasters due to smaller scale and nature of the incident. However, the findings of this study can predict what challenges and patient outcomes can occur in case of any event causing exposure on a larger scale in a metropolitan city such as Karachi in a lower-middle income country. Although many case studies have been published on accidental chlorine exposure due to swimming pool accidents and household exposure, there are few studies on large scale industrial exposure.

All patients in our study were males and adults. This accounts for the relatively better outcomes in our study since children are reported to be more severely affected by chlorine exposure(14). This was observed in various studies as children had a higher hospitalization rate and longer hospital stay(14).

The presenting features and symptoms of the patients were similar to those observed in other observational studies. Cough and dyspnea were the most common symptoms observed in our study. Sever et al.(11) in Turkey reported similar outcomes and clinical features in 39 patients presenting to emergency department after accidental chlorine gas exposure. Cough and dyspnea were seen in 64.1% and 30.8% of the patients, respectively. Sever et al.(11) also reported these as the most common symptoms. In a systematic review of over 37 incidents of accidental chlorine exposure in civilians (n=1566), cough and dyspnea were also the most common presenting features, observed in 29% and 22% patients, respectively(2). In contrast, Kim et al. reported headache as the most common symptom observed in patients exposed(14) and eye irritation with sneezing was common in patients in a study by Ammoura et al. in Jordan(7). Physical signs showed significant correlation with outcomes in a study by Khilji et al. from Oman(9). However, tachycardia was the only finding significantly associated with oxygen or NIV requirement in our study. 

30.8% were asymptomatic and physical examination was unremarkable in the majority (64.1%) of patients and the most common physical examination finding was tachypnea in a study on evaluation of 39 cases of chlorine exposure by Sever et al. in Turkey(11). However, the majority of patients had an abnormal physical examination in our study; tachypnea was the most common physical sign, found in 29 (76.3%) patients. All 71 patients (100%) had presented with respiratory complains after the train derailment incident in Graniteville, California(12). On physical exam wheeze (46%) and crackles (29%) were the most common findings; tachypnea was observed in 46%(12). Gülolu et al. (8)from Turkey also reported 10.4% asymptomatic subjects and normal physical examination in the majority (71.6%) of patients. 

Only one patient (2.6%) had abnormal x-ray in the study by Sever et al.(11); in contrast to 15.6% patients having abnormal chest x-ray findings in our study. Non-invasive ventilation was required in 9 (27.3%) patients in our study, in comparison, no patient in their study required non-invasive or invasive mechanical ventilation. Chest x-rays were done in 71.7% (76/106) and x-rays were pathological in only 6 (5.7%). In contrast, 40/71 (57%) of the hospitalized patients developed abnormal findings after the Graniteville train derailment incident(12), most of these x-ray changes occurred on the first day of exposure and infiltrates were frequent findings. The role of chest-x-ray in management after chlorine exposure is controversial; most recommend it in cases of pulmonary edema or acute respiratory distress syndrome (ARDS), however, others recommend a baseline chest x-ray in all patients with exposure to assess radiological progression(3).

Kim et al.(14) did not report any laboratory abnormalities among the 209 non-hospitalized patients after chlorine exposure. Arterial Blood Gas (ABG) assessments were done in only 55 (77%) cases in a study by Van Sickle (12). Mean pH was 7.32, which was similar to7.35 reported in our study. 25 (45%) patients had respiratory acidosis in their study as compared to 30% in our study and 18 (10%) had hypoxemia in their study as compared to 69.2% of our patients in whom ABGs were assessed. Similarly, ABG assessments were not done in 85/106 (80.2%) patients in a study by Gülolu et al.(8) Among those with ABG assessments,results were within normal ranges in 13/21 (61.9%), 4/21 (19.04%) had hypoxia, 3/21 (14.2%) patients had hypercarbia and one patient had hypocarbia and respiratory alkalosis.

In a systematic review of 36 studies(2), overall death rate in 1566 individuals was 0.6%, all of the 9 patients who had died in that systematic review belonged to the study on Graniteville train derailment incident(12).There were four cases of spontaneous pneumomediastinum (0.3%)(2). Our study had one case of pneumomediastinum, and no patient died. Although this study did not assess lung function tests, chlorine gas exposure can result in long-term sequela due to reduction in lung function and this was reported in various studies(10, 15, 16).

Masoumi et al. from neighboring Iran reported significant problems, which were encountered after chlorine gas leakage in Dezful, Iran(10). Prevention of these events requires environmental regulations, chlorine safety-related guidelines implementation and pilot safety studies in industrial installations. Emergency response systems in hospitals for rapid implementation during these disasters can ensure effective management.

**Table 1:** Clinical and laboratory findings of studied cases

**Variables**	**Values**	
**Symptoms**		
Dyspnea	33 (86.8)	
Cough	27 (71.0)	
Cough and dyspnea	25 (65.7)	
Chest pain	17 (44.7)	
Headache	5 (13.2)	
Sore throat	4 (10.5)	
Nausea	4 (10.5)	
Vomiting	2 (5.3)	
**Physical examination**		
Tachycardia	19 (50.0)	
Tachypnea	29 (76.3)	
Expiratory wheeze	4 (10.5)	
Bilateral lung crepitation	7 (18.4)	
**Arterial Blood gas (n = 13)**		
Mean pH	7.35 ± 0.14	
Median Po2 mmHg (IQR)	64 (55.5 – 94.0)	
Median PCo2 mmHg (IQR)	41.5 (38.9 - 46.75)	
**Laboratory parameters (n=32)**		
Hemoglobin(g/dl)	14.65± 0.99	
White cell count(x10^9)	13.89± 4.64	
Neutrophils (%)	79.43 ±12.01	
Neutrophilia	17 (44.7)	
Lactate(n=13)	2.18± 1.27	
Sodium(mmol/l)	139.28 ± 2.27	
Potassium(mmol/l)	4.00 ± 0.38	
Bicarbonate(mmol/l)	25.15 ± 2.74	
Chloride(mmol/l)	104.56 ± 1.74	
Troponin(ng/ml)	0.0072 ± 0.0036	

Data are presented as mean ± standard deviation or frequency (%). Complete baseline laboratory data were available for 32 patients who were shifted to emergency room long stay area; whereas this was missing in other patients as they were observed clinically and discharged from short stay area.

**Table 2:** Management and outcomes of studied patients

**Variables**	Values	
**Length of stay (days)**		
Mean ± SD	1.55 ± 1.58	
≤ 1*	29 (76.3)	
2	6 (15.8)	
≥ 4	3 (7.9)	
**Disposition**		
Ward	24 (63.2)	
Emergency department	7 (18.4)	
Special care unit	6 (15.8)	
Intensive care unit	1 (2.6)	
**Management**		
Salbutamol nebulization	32 (84.2)	
Ipratropium nebulization	34 (89.5)	
Systemic steroids	13 (34.2)	
Sodium bicarbonate nebulization	2 (5.3)	
Beclomethasone nebulization	5 (13.2)	
Supplemental oxygen	18 (47.4)	
Non-invasive ventilation	9 (23.7)	
Mechanical ventilation	1 (2.6)	

Data are presented as mean ± standard deviation (SD) or frequency (%). *: 10 (26.3%) were hospitalized for < 6 hours.

**Table 3:** Association between some clinical features and need for oxygen supplementation and non-invasive ventilation (NIV)

**Variable**	**Total (n)**	**Oxygen **	**P**	**NIV **	**P**
**Age (years)**					
≥ 40	6	4 (66.7)	0.33	3 (50.0)	0.13
< 40	32	15 (46.9)		6 (18.0)	
**Chest x-ray abnormality**					
Yes	5	5 (100.0)	0.049	4 (80.0)	0.013
No	27	14 (51.9)		5 (18.5)	
**Tachypnea**					
Yes	29	16 (55.2)	0.22	9 (31.0)	0.061
No	9	3 (33.3)		0	
**Tachycardia**					
Yes	19	13 (68.4)	0.033	8 (42.1)	0.019
No	19	6 (31.6)		1 (5.3)	

Data are presented as frequency (%).

## 5. Limitation

Limitations of our study include being single-centered and retrospective study design. Exact number of exposed people is not known and patients presented to various tertiary care hospitals in Karachi after the incident; therefore, outcomes of those patients are not known. Distance from leakage site; exposure duration, and concentration of chlorine was not documented properly. Long-term follow up of patients was not done to assess for persistence of symptoms. Moreover, lung functions test during admission and post-admission were not done to assess bronchial hyperactivity. Our center is a large tertiary care private hospital; therefore, the results of the study cannot be generalized to other centers in the country.

## 6. Conclusion:

The majority of patients presenting with acute chlorine gas exposure showed good clinical outcomes and rapid recovery; however, a high index of suspicion and vigilance should be maintained for complications such as pneumomediastinum and acute respiratory distress syndrome in these patients. Prevention of these events requires environmental regulations, chlorine safety-related guidelines implementation, and pilot safety studies in industrial installations. Moreover, in case of these incidents the duration of exposure, proximity, and concentration of the toxin should be documented.

## 7. Declarations:

### 7.1 Conflict of interest:

Authors declare that there is no conflict of interest.

### 7.2 Acknowledgement:

None.

### 7.3 Funding:

None.

### 7.4 Authors’ contributions:

Taymmia Ejaz, Safia Akhlaq and Adil Aziz designed the study. Taymmia Ejaz and Safia Akhlaq participated in acquisition of data. Sheema Saadia and Arslan Ahmed analyzed the data. Adil Aziz and Arslan Ahmed reviewed results and carried out interpretation of data. Taymmia Ejaz and Sheema Saadia wrote the initial draft and others revised the manuscript critically. All authors approved final version of the manuscript to be published and are accountable for all aspects of the work.

## References

[1] Carlisle M, Lam A, Svendsen ER, Aggarwal S, Matalon S (2016). Chlorine-induced cardiopulmonary injury. Annals of the New York Academy of Sciences..

[2] Govier P, Coulson JM (2018). Civilian exposure to chlorine gas: A systematic review. Toxicol Lett..

[3] Zellner T, Eyer F (2020). Choking agents and chlorine gas - History, pathophysiology, clinical effects and treatment. Toxicol Lett..

[4] Huynh Tuong A, Despreaux T, Loeb T, Salomon J, Megarbane B, Descatha A (2019). Emergency management of chlorine gas exposure - a systematic review. Clin Toxicol (Phila)..

[5] Balte PP, Clark KA, Mohr LC, Karmaus WJ, Van Sickle D, Svendsen ER (2013). The Immediate Pulmonary Disease Pattern following Exposure to High Concentrations of Chlorine Gas. Pulm Med..

[6] Hoyle GW, Svendsen ER (2016). Persistent effects of chlorine inhalation on respiratory health. Annals of the New York Academy of Sciences..

[7] Ammoura AM, S.Aqqad A (2004). Chlorine Gas Poisoning. Journal of The Royal Medical Services.

[8] Güloğlu C, Kara İH, Erten PG (2002). Acute Accidental Exposure to Chlorine Gas in the Southeast of Turkey: A study of 106 Cases. Environmental Research..

[9] Khilji MF (2021). Clinical Presentations and Outcomes of Industrial Chlorine Gas Exposure Incidence in Oman. Prehospital and Disaster Medicine..

[10] Masoumi G, Maniey M, Aghababaeian H, Ostadtaghizadeh A, Araghi Ahvazi L (2020). Lessons Learned From a Chlorine Gas Leakage in Dezful City, Iran. Disaster Medicine and Public Health Preparedness.

[11] Sever M, Mordeniz C, Sever F, Dokur M (2009). Accidental Chlorine Gas Intoxication: Evaluation of 39 Patients. Journal of Clinical Medicine Research.

[12] Van Sickle D, Wenck MA, Belflower A, Drociuk D, Ferdinands J, Holguin F (2009). Acute health effects after exposure to chlorine gas released after a train derailment. Am J Emerg Med..

[13] Bhatti MW 137 faint in Port Qasim industrial gas leakage. The News.

[14] Kim J-A, Yoon S-Y, Cho S-Y, Yu J-H, Kim H-S, Lim G-I (2014). Acute health effects of accidental chlorine gas exposure. Ann Occup Environ Med..

[15] Christensen BE, Duncan MA, King SC, Hunter C, Ruckart P, Orr MF (2016). Challenges During a Chlorine Gas Emergency Response. Disaster Med Public Health Prep..

[16] Clark KA, Karmaus WJ, Mohr LC, Cai B, Balte P, Gibson JJ (2016). Lung Function before and after a Large Chlorine Gas Release in Graniteville, South Carolina. Ann Am Thorac Soc..

